# Hyperammonemia syndrome in a liver transplant recipient due to *Ureaplasma parvum*: a case report

**DOI:** 10.1093/jscr/rjaf1093

**Published:** 2026-01-22

**Authors:** Shreya Tamma, Eric Dorris, Emma Hills, Sarah G Van Winkle, Rayhan Jhanji, John Goss, Christine O’Mahony, Nhu T N Galván, Abbas Rana, Chun-Sing Huang

**Affiliations:** School of Medicine, Baylor College of Medicine, One Baylor Plaza, Houston, TX 77030, United States; School of Medicine, Baylor College of Medicine, One Baylor Plaza, Houston, TX 77030, United States; Division of Abdominal Transplantation, Michael E. DeBakey Department of General Surgery, One Baylor Plaza, Houston, TX 77030, Baylor College of Medicine, Houston, TX, United States; School of Medicine, Baylor College of Medicine, One Baylor Plaza, Houston, TX 77030, United States; School of Medicine, Baylor College of Medicine, One Baylor Plaza, Houston, TX 77030, United States; Division of Abdominal Transplantation, Michael E. DeBakey Department of General Surgery, One Baylor Plaza, Houston, TX 77030, Baylor College of Medicine, Houston, TX, United States; Division of Abdominal Transplantation, Michael E. DeBakey Department of General Surgery, One Baylor Plaza, Houston, TX 77030, Baylor College of Medicine, Houston, TX, United States; Division of Abdominal Transplantation, Michael E. DeBakey Department of General Surgery, One Baylor Plaza, Houston, TX 77030, Baylor College of Medicine, Houston, TX, United States; Division of Abdominal Transplantation, Michael E. DeBakey Department of General Surgery, One Baylor Plaza, Houston, TX 77030, Baylor College of Medicine, Houston, TX, United States; Division of Abdominal Transplantation, Michael E. DeBakey Department of General Surgery, One Baylor Plaza, Houston, TX 77030, Baylor College of Medicine, Houston, TX, United States

**Keywords:** hyperammonemia syndrome, orthotopic liver transplantation, *Ureaplasma* parvum, infectious complications, solid organ transplant

## Abstract

Hyperammonemia syndrome is a rare, life-threatening complication following orthotopic liver transplantation. While most cases result from graft dysfunction, metabolic disorders, or portosystemic shunting, infectious etiologies are infrequently recognized. *Ureaplasma* species, typically urogenital commensals, have recently emerged as important pathogens. We describe the first reported case of *Ureaplasma parvum*-associated hyperammonemia syndrome following isolated orthotopic liver transplantation. A 63-year-old man developed severe hyperammonemia and encephalopathy on postoperative day 14, despite preserved graft function and embolization of a large splenorenal shunt. In the absence of other causes and with rapid clinical decline, empiric doxycycline was initiated. *U. parvum* was subsequently detected via bronchoalveolar lavage and plasma cell-free DNA testing. Continued doxycycline led to normalization of ammonia levels and neurologic recovery. This case underscores the importance of considering *Ureaplasma* infection in post-orthotopic liver transplantation hyperammonemia and supports early empiric antimicrobial therapy to prevent fatal outcomes.

## Introduction

Hyperammonemia syndrome (HS) is a rare but potentially fatal complication following orthotopic liver transplantation (OLT). Defined by markedly elevated serum ammonia, HS can precipitate seizures, cerebral edema, and death. Common etiologies include primary graft dysfunction (PGD), unmasked urea cycle disorders, and portosystemic shunting, whereas infectious causes are less often recognized [1].

*Ureaplasma* species are urease-producing commensal organisms of the urogenital tract that have emerged as an important infectious cause of HS in immunosuppressed hosts. By hydrolyzing urea to ammonia, disseminated *Ureaplasma* infection can overwhelm hepatic clearance, even in the setting of preserved graft function [2–4]. Previously reported cases occurred mainly in lung and kidney transplants, with only one in a combined liver-kidney recipient [3–5].

Here, we describe the first case of *U. parvum*-associated HS following isolated OLT. Our report emphasizes the need to recognize treatable infectious causes of post-OLT HS, particularly in a differential often dominated by primary hepatic or metabolic etiologies. As *Ureaplasma* is undetectable by standard cultures, molecular diagnostics are required, and empiric antibiotics may be lifesaving when initiated early [2].

## Case report

A 63-year-old man with nonalcoholic steatohepatitis cirrhosis, type 2 diabetes mellitus, and hypertension underwent OLT in May 2025. The donor was a 24-year-old male with isolated head trauma and a liver biopsy revealing 21%–30% macrosteatosis. The graft underwent normothermic machine perfusion with normal flows and lactate clearance. The operation was uncomplicated.

**Figure 1:**
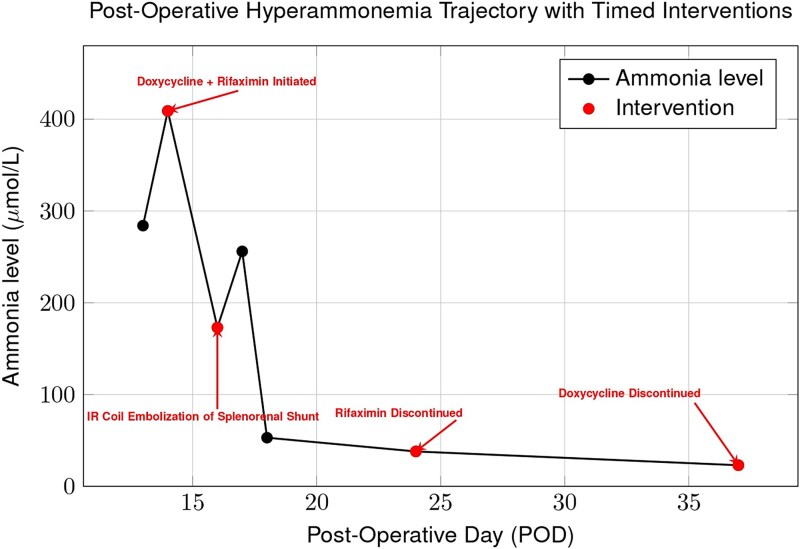
Trend of plasma ammonia levels post-OLT. Despite initiation of doxycycline and rifaximin, ammonia rose after coil embolization of a splenorenal shunt, indicating that portosystemic shunting was not the sole cause. Levels subsequently declined with continued doxycycline, supporting *Ureaplasma parvum* infection as the primary driver of hyperammonemia.

**Figure 2:**
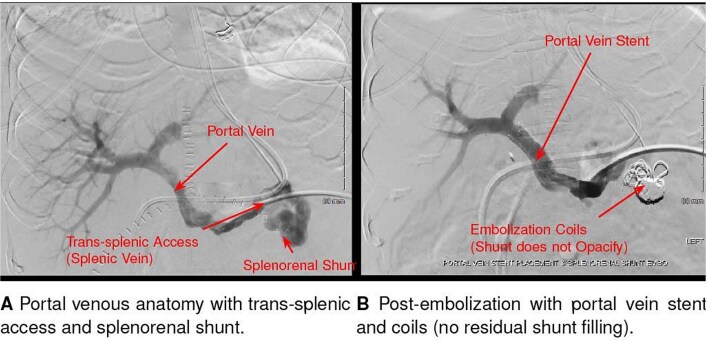
Interventional radiology image of coil and liquid embolization of a large splenorenal shunt with concurrent portal vein stent placement, performed for refractory hyperammonemia after liver transplantation.

On POD11, cholestatic liver test elevation prompted endoscopic retrograde cholangiopancreatography; after sphincterotomy and placement of two stents, values improved. On POD14, the patient developed acute altered mental status. The ammonia level was 409 μmol/L, and intermittent hemodialysis was initiated. Empiric doxycycline and rifaximin were started the same day (Fig. 1). A non-contrast head CT was unremarkable. Abdominal ultrasound and CT showed preserved hepatic arterial and portal venous flow, as well as a large preserved splenorenal shunt seen prior to OLT and mild stenosis of the portal vein. On POD16, interventional radiology embolized the splenorenal shunt and stented the portal vein, with no improvement in ammonia levels following these interventions (Fig. 2). An unmasked urea cycle disorder was also considered to explain hyperammonemia but deemed unlikely given the donor history. The patient was maintained on standard immunosuppression with daily tacrolimus and prednisone.

Earlier broad-spectrum antibiotic therapy with meropenem and vancomycin lacked activity against *Ureaplasma*; after empiric doxycycline was started on POD14, ammonia levels declined to <100 μmol/L and normalized by POD18 (Fig. 1). A bronchoalveolar lavage (BAL) obtained on POD17 returned positive by polymerase chain reaction (PCR) for *U. parvum* on POD20. Plasma cell-free DNA sequencing (Karius test), collected the same day, returned concordant results on POD21. Rifaximin was discontinued, and doxycycline was continued to complete a 23-day course.

Neurologic recovery paralleled biochemical improvement. Sedation was stopped POD19; brain MRI on POD 22 was normal. The patient followed commands by POD 23 and was extubated POD 24. EEG on POD 25 showed diffuse slowing without seizures. He remained hemodynamically stable with no recurrent hyperammonemia. Blood and urine cultures were negative except for early *Candida tropicalis* colonization. The patient was discharged POD 58 and continues to do well on follow-up.

## Discussion

This case highlights the diagnostic complexity of hyperammonemia after OLT, particularly when graft function is preserved. Hyperammonemia in post-transplant patients can precipitate rapid cerebral edema, seizures, coma, and death, so empiric management must begin as the diagnostic evaluation proceeds [1, 6–8]. Etiologies include portosystemic shunting, inborn errors of metabolism such as urea cycle disorders in the donor graft, and infections with urease-producing organisms such as *Ureaplasma* species.

Given the rapid progression and severity of neurologic symptoms, empiric antimicrobial therapy with doxycycline was initiated prior to microbiologic confirmation and was followed by an initial decline in ammonia levels. In contrast, embolization of the large spontaneous splenorenal shunt produced no immediate improvement, highlighting that abnormalities in vascular flow alone could not explain the clinical course. While this temporal pattern supports a role for *Ureaplasma* infection, causality cannot be definitively established and a multifactorial etiology remains possible [5, 9, 10]. The turning point came with the detection of *U. parvum* via both BAL and cell-free DNA testing on plasma, which were performed as part of the broad infectious diseases workup. BAL confirmed local pulmonary involvement, while Karius testing demonstrated systemic dissemination, together strengthening the diagnosis and justifying continuation of targeted therapy [5, 11]. The observed rapid neurologic improvement underscores the potential reversibility of *Ureaplasma*-induced hyperammonemia with appropriate antibiotics.

*Ureaplasma* is a fastidious, urease-producing organism that cannot be detected on routine bacterial cultures [12]. In transplant recipients, advanced molecular diagnostics such as PCR or next-generation sequencing are often required for identification. Infections most commonly occur in lung transplant patients but have been reported in kidney and, rarely, liver transplant recipients [3, 4, 11]. In immunocompromised patients, disseminated infection can overwhelm hepatic ammonia clearance due to potent urease activity, resulting in rapid and profound hyperammonemia with risk of seizures, coma, and death [2, 3, 9]. Recommended therapy includes tetracyclines (such as doxycycline), macrolides, or fluoroquinolones. Empiric treatment for 2–3 weeks is reasonable in suspected cases, as delays in therapy worsen outcomes.

A donor-derived urea cycle disorder was also considered early in the evaluation. However, urea cycle disorder transmission through liver grafts is exceedingly rare [13], and the donor’s history did not suggest a previously undiagnosed disorder. Moreover, the elevated and fluctuating ammonia pattern in our patient, despite dialysis, phenyl benzoate, and arginine, was atypical for a urea cycle disorder, which usually shows rapid, sustained improvement with these interventions [13–15]. While confirmatory genetic studies remain prudent, the lack of response to standard metabolic therapies and the subsequent clinical improvement with doxycycline make *Ureaplasma* infection the more compelling explanation.

In summary, this case illustrates the importance of considering *Ureaplasma* infection in the differential diagnosis of hyperammonemia after OLT, particularly when routine workup is unrevealing or neurologic decline is out of proportion to liver dysfunction. While interventional approaches such as shunt embolization may correct flow-dependent causes, they will not resolve hyperammonemia driven by systemic ammonia production from infection. Prompt recognition and initiation of targeted antimicrobial therapy are therefore essential and can be lifesaving.

## Data Availability

The data supporting the findings of this study are contained within the article.
